# Supplementary material for: Binge-pattern alcohol consumption and genetic risk as determinants of alcohol-related liver disease
Source: Nat Commun. 2023 Dec 14;14:8041. doi: 10.1038/s41467-023-43064-x (PMC10721893; doi:10.1038/s41467-023-43064-x)
Supplement: Supplementary file 1 — Supplementary Information [file 41467_2023_43064_MOESM1_ESM.pdf]

Supplementary Information

Binge-Pattern Alcohol Consumption and Genetic Risk Factors as Determinants of Risk of Alcohol-Related Liver Disease

Supplementary Notes

**Supplemental analyses**	
**Supplemental Figure 1:** Distribution of PRS for ARLD	Page 2
**Supplemental Table 1:** Baseline characteristics by alcohol consumption group in UK Biobank	Page 3
**Supplemental Table 2:** Risk of advanced ARLD with PRS	Page 5
**Supplemental Table 3:** Two-way additive interaction between alcohol consumption and PRS	Page 6
**Supplemental Table 4:** Two-way additive interaction of diabetes with alcohol consumption and PRS	Page 7
**Supplemental analyses using competing risk regression models**	
**Supplemental Table 5:** Risk of advanced ARLD with alcohol consumption and PRS – competing risk regression models	Page 8
**Supplemental Table 6:** Risk of ARC by alcohol consumption and PRS – competing risk regression models	Page 9
**Supplemental Table 7:** Risk of ARC by diabetes and (a) alcohol consumption and (b) PRS – competing risk regression models	Page 10
**Supplemental Table 8:** Two-way additive interaction of alcohol consumption, PRS and diabetes – competing risk regression models	Page 11
**Supplemental analyses using imputed data**	
**Supplemental Table 9:** Risk of advanced ARLD with alcohol consumption and PRS using imputed data	Page 12
**Supplemental Table 10:** Risk of ARC by alcohol consumption and PRS using imputed data	Page 13
**Supplemental Table 11:** Risk of ARC by diabetes and (a) alcohol consumption and (b) PRS using imputed data	Page 14
**Supplemental Table 12:** Two-way additive interaction of alcohol consumption, PRS and diabetes using imputed data	Page 15
**Supplemental Table 13:** Three-way additive interaction of alcohol consumption, PRS and diabetes using imputed data	Page 16
**Replication analyses in the China Kadoorie Biobank (CKB) cohort**	
**Supplemental Table 14a:** Baseline characteristics of male, current weekly drinkers in CKB by disease status	Page 17
**Supplemental Table 14b:** Baseline characteristics of male, current weekly drinkers in CKB by alcohol consumption group	Page 18
**Supplemental Table 15:** Risk of advanced ARLD with alcohol consumption – replication analyses in CKB	Page 20
**Supplemental Table 16:** Risk of ARC by diabetes and alcohol consumption – replication analyses in CKB	Page 21
**Supplemental Table 17:** Two-way additive interaction of alcohol consumption and diabetes – replication analyses in CKB	Page 21
**Supplemental Table 18:** Risk of advanced ARLD with per 1 SD increase in PRS in CKB	Page 22
**Cross-validation analyses using UKB sub-samples**	
**Supplemental Table 19:** Risk of advanced ARLD with alcohol consumption and PRS – cross-validation using sub-samples	Page 23
**Supplemental Table 20:** Risk of ARC by alcohol consumption and PRS – cross-validation using sub-samples	Page 24
**Supplemental Table 21:** Risk of ARC by diabetes and (a) alcohol consumption and (b) PRS – cross-validation using sub-samples	Page 25
**Supplemental Table 22:** Two-way additive interaction of alcohol consumption, PRS and diabetes – cross-validation using sub-samples	Page 26
**Supplemental Table 23:** Three-way additive interaction of alcohol consumption, PRS and diabetes – cross-validation using sub-samples	Page 27

Supplemental analyses

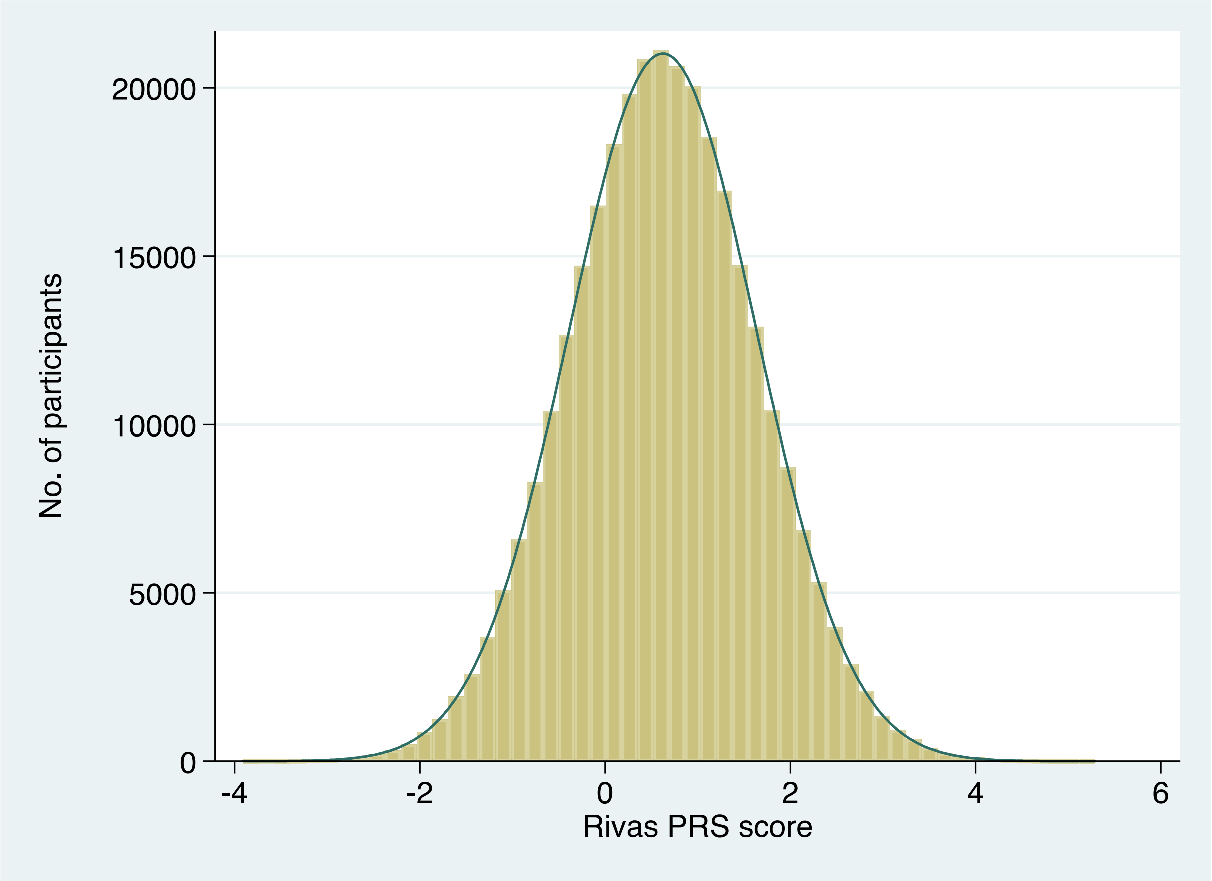

Supplemental Figure 1: Distribution of polygenic risk score (from Sinnott-Armstrong et al[25].) for alcohol-related liver disease

Supplemental Table 1: Baseline characteristics in UK Biobank study by alcohol consumption group*

	Total (N=312599)	Within daily limit (n=61742)	Above daily limit but below binge (n=132012)	Binge (n=71529)	Heavy Binge (n=47316)	p value ****
Age, years **	69.4 (8.0)	71.0 (7.8)	69.7 (7.9)	68.7 (8.0)	67.4 (8.0)	<0.001
Weekly alcohol intake, units **	23.4 (20.0)	9.3 (5.8)	18.8 (10.6)	29.6 (16.9)	45.7 (31.3)	<0.001
Male	159826 (51.1)	27108 (43.9)	64092 (48.6)	39030 (54.6)	29596 (62.5)	<0.001
Ethnicity, white	294816 (94.3)	57227 (92.7)	124275 (94.1)	68078 (95.2)	45236 (95.6)	<0.001
Smoking status						
Never	162688 (52.0)	38219 (61.9)	72294 (54.8)	33456 (46.8)	18719 (39.6)	
Ex-smoker	119065 (38.1)	19921 (32.3)	49534 (37.5)	29710 (41.5)	19900 (42.1)	
Current smoker	30846 (9.9)	3602 (5.8)	10184 (7.7)	8363 (11.7)	8697 (18.4)	<0.001
Physical activity ***						
None	108143 (34.6)	22590 (36.6)	44416 (33.6)	24379 (34.1)	16758 (35.4)	
1-2 days	98897 (31.6)	20557 (33.3)	43811 (33.2)	21828 (30.5)	12701 (26.8)	
3+ days	105559 (33.8)	18595 (30.1)	43785 (33.2)	25322 (35.4)	17857 (37.7)	<0.001
Frequency of drinking						
Daily or almost daily	92532 (29.6)	21356 (34.6)	40799 (30.9)	19671 (27.5)	10706 (22.6)	
3-4 times a week	105170 (33.6)	18127 (29.4)	49923 (37.8)	24403 (34.1)	12717 (26.9)	
1-3 times a week	114897 (36.8)	22259 (36.1)	41290 (31.3)	27455 (38.4)	23893 (50.5)	<0.001
Alcohol type						
Mixed	161900 (51.8)	33464 (54.2)	69444 (52.6)	36672 (51.3)	22320 (47.2)	
Wine only	8497 (2.7)	2041 (3.3)	3882 (2.9)	1835 (2.6)	739 (1.6)	
Beer only	93289 (29.8)	13553 (22.0)	37159 (28.1)	23705 (33.1)	18872 (39.9)	
Spirits only	48913 (15.6)	12684 (20.5)	21527 (16.3)	9317 (13.0)	5385 (11.4)	<0.001
Drinking with meals						
Not with meals	63648 (20.4)	11330 (18.4)	20774 (15.7)	16121 (22.5)	15423 (32.6)	
With meals	136797 (43.8)	34485 (55.9)	64887 (49.2)	26203 (36.6)	11222 (23.7)	
It varies	112154 (35.9)	15927 (25.8)	46351 (35.1)	29205 (40.8)	20671 (43.7)	<0.001
PRS Score						
Low	63549 (20.3)	13376 (21.7)	27543 (20.9)	14139 (19.8)	8491 (17.9)	
Middle	188524 (60.3)	37176 (60.2)	79613 (60.3)	43083 (60.2)	28652 (60.6)	
High	60526 (19.4)	11190 (18.1)	24856 (18.8)	14307 (20.0)	10173 (21.5)	<0.001
BMI, kg/m2						
<18.5	1367 (0.4)	457 (0.7)	578 (0.4)	203 (0.3)	129 (0.3)	
18.5-24.9	107985 (34.5)	25820 (41.8)	49111 (37.2)	21624 (30.2)	11430 (24.2)	
25-29.9	138404 (44.3)	25349 (41.1)	58173 (44.1)	33182 (46.4)	21700 (45.9)	
≥30	64843 (20.7)	10116 (16.4)	24150 (18.3)	16520 (23.1)	14057 (29.7)	<0.001

Prevalent diabetes	11842 (3.8)	2280 (3.7)	4622 (3.5)	2810 (3.9)	2130 (4.5)	<0.001
Advanced ARLD	782 (0.3)	48 (0.1)	161 (0.1)	209 (0.3)	364 (0.8)	<0.001
ARC	734 (0.2)	47 (0.1)	151 (0.1)	194 (0.3)	342 (0.7)	<0.001
AH	136 (0.04)	4 (0.01)	23 (0.02)	36 (0.05)	73 (0.15)	<0.001

Values are numbers (percentages) unless otherwise indicated

* Based on average daily alcohol consumption: within daily limit (<24g for women, <32g for men), above daily limit but below binge (24-48g for women, 32-64g for men), binge (48-72g for women, 64-96g for men) and heavy binge (>=72g for women, >=96g for men)

** Mean (standard deviation)

*** Physical activity based on the number of days engaged in vigorous activity for 10 minutes or more

**** Two-sided p values from one-way ANOVA or chi-square test (Fisher's exact test for cells with <5 observations) to compare the differences across binge groups

Supplemental Table 2: Hazard ratios and 95% confidence intervals for risk of advanced ARLD associated with polygenic risk

		ARC				AH			
	**Participants (N)**	**Cases (n)**	**HR**	**95% CI**	**p value**	**Cases (n)**	**HR**	**95% CI**	**p value**
Polygenic risk score category									
Low group	63,549	71	Ref			20	Ref		
Middle group	188,524	373	1.60	(1.24, 2.07)	<0.001	77	1.13	(0.69, 1.86)	0.622
High group	60,526	290	3.51	(2.70, 4.55)	<0.001	39	1.60	(0.93, 2.76)	0.088
Polygenic risk score per SD *	312,599	734	1.67	(1.55, 1.80)	<0.001	136	1.32	(1.12, 1.57)	<0.001

HRs were derived from Cox regression with adjustment for sex, age, ethnicity, Townsend deprivation index at recruitment, physical activity, smoking, total weekly alcohol intake, beverage type, drinking with/without meal, daily alcohol consumption group, diabetes, BMI, first 10 principal genetic components, and genotyping array; p values were calculated using the two-sided Wald test

* One SD corresponded to 1.015

Supplemental Table 3: Two-way additive interaction between alcohol consumption and PRS group

	Participants (N)	ARC cases (n)	RERI	95% CI	p value	AP	95% CI	p value	S	95% CI	p value
Above daily limit but below binge # Mid PRS	79613	70	-0.34	(-1.73, 1.05)	0.632	-0.38	(-1.70, 0.94)	0.571	-0.47	(-8.28, 7.33)	0.711
Above daily limit but below binge # High PRS	24856	62	-1.31	(-4.09, 1.46)	0.354	-0.56	(-1.55, 0.43)	0.265	0.51	(0.05, 0.96)	0.033
Binge # Mid PRS	43083	106	-0.00	(-1.18, 1.17)	0.994	-0.00	(-0.98, 0.98)	0.994	0.98	(-4.68, 6.63)	0.994
Binge # High PRS	14307	70	-1.67	(-5.04, 1.70)	0.332	-0.58	(-1.61, 0.45)	0.270	0.53	(0.06, 1.00)	0.049
Heavy binge # Mid PRS	28652	175	2.26	(-0.08, 4.60)	0.058	***0.32***	***(0.03, 0.61)***	***†0.029***	1.60	(0.68, 2.51)	0.200
Heavy binge # High PRS	10173	138	***6.07***	***(0.20, 11.94)***	***†0.043***	***0.47***	***(0.25, 0.70)***	***†<0.001***	2.06	(1.00, 3.12)	0.050

Based on hazard ratio estimates derived from Cox regression with adjustment for total weekly alcohol intake, BMI, diabetes, sex, age, ethnicity, Townsend deprivation index, physical activity, smoke, alcohol type, drinking with/without meals, genotyping array, first 10 ancestry principal components; p values were calculated using the two-sided Wald test

†RERI>0, AP>0 or S>1 indicates significant additive interaction

Supplemental Table 4: Two-way additive interaction of diabetes with alcohol consumption and PRS group

	Participants (N)	ARC cases (n)	RERI	95% CI	p value	AP	95% CI	p value	S	95% CI	p value
Diabetes # Above daily limit but below binge *	4622	21	-0.69	(-2.85, 1.48)	0.533	-0.43	(-1.86, 0.99)	0.548	0.46	(-0.46, 1.38)	0.247
Diabetes # Binge *	2810	27	-0.63	(-3.11, 1.85)	0.619	-0.30	(-1.53, 0.92)	0.631	0.63	(-0.36, 1.62)	0.470
Diabetes # Heavy binge *	2130	48	***4.69***	***(0.50, 8.88)***	***†0.028***	***0.43***	***(0.16, 0.71)***	***†0.002***	1.92	(0.84, 3.00)	0.096
Diabetes # Mid PRS **	7080	48	0.79	(-1.24, 2.81)	0.448	0.20	(-0.29, 0.69)	0.424	1.36	(0.17, 2.56)	0.552
Diabetes # High PRS **	2600	46	***4.83***	***(0.99, 8.67)***	***†0.014***	***0.46***	***(0.21, 0.70)***	***†<0.001***	2.02	(0.95, 3.10)	0.063

Based on hazard ratio estimates derived from Cox regression, with two-sided p values computed using the Wald test
*Adjusted for total weekly alcohol intake, BMI, sex, age, ethnicity, Townsend deprivation index, physical activity, smoke, alcohol type, drinking with/without meals, PRS group, genotyping array, first 10 ancestry principal components
**Adjusted for total weekly alcohol intake, daily alcohol consumption group, BMI, sex, age, ethnicity, Townsend deprivation index, physical activity, smoke, alcohol type, drinking with/without meals, genotyping array, first 10 ancestry principal components
†RERI>0, AP>0 or S>1 indicates significant additive interaction

Analyses using competing risk regression models

Supplemental Table 5: Hazard ratios for risk of advanced ARLD associated with alcohol consumption and polygenic risk – competing risk regression models (N=312,599)

	ARC (n=734)			AH (n=136)		
	**HR**	**95% CI**	**p value**	**HR**	**95% CI**	**p value**
Alcohol consumption group *						
Below daily limit	Ref					
Above daily limit but below binge	1.33	(0.95, 1.85)	0.095	2.38	(0.82, 6.90)	0.110
Binge	2.34	(1.69, 3.26)	<0.001	5.11	(1.82, 14.38)	0.002
Heavy binge	3.79	(2.70, 5.31)	<0.001	9.23	(3.31, 25.77)	<0.001
Polygenic risk score category **						
Low group	Ref			Ref		
Middle group	1.60	(1.22, 2.09)	0.001	1.13	(0.68, 1.87)	0.632
High group	3.45	(2.63, 4.52)	<0.001	1.58	(0.91, 2.74)	0.106

HRs were derived from Cox regression, with two-sided p values computed using the Wald test
*Adjusted for sex, age, ethnicity, Townsend deprivation index at recruitment, physical activity, smoking, total weekly alcohol intake, beverage type, drinking with/without meal, diabetes, and BMI
**Adjusted for sex, age, ethnicity, Townsend deprivation index at recruitment, physical activity, smoking, total weekly alcohol intake, beverage type, drinking with/without meal, daily alcohol consumption group, diabetes, BMI, first 10 principal genetic components, and genotyping array

Supplemental Table 6: Hazard ratios for risk of alcohol-related cirrhosis by alcohol consumption and polygenic risk group – competing risk regression models (N=312,599)

	ARC (n=734)		
	**HR**	**95% CI**	**p value**
Below daily limit			
Low PRS	Ref		
Mid PRS	1.52	(0.58, 4.02)	0.398
High PRS	4.31	(1.61, 11.53)	0.004
Above daily limit but below binge			
Low PRS	1.60	(0.59, 4.30)	0.353
Mid PRS	1.96	(0.79, 4.87)	0.149
High PRS	5.18	(2.07, 12.95)	<0.001
Binge			
Low PRS	2.19	(0.81, 5.96)	0.124
Mid PRS	4.04	(1.63, 10.02)	0.003
High PRS	7.30	(2.91, 18.30)	<0.001
Heavy binge			
Low PRS	3.47	(1.29, 9.35)	0.014
Mid PRS	5.86	(2.36, 14.56)	<0.001
High PRS	12.41	(4.96, 31.05)	<0.001

HRs were derived from Cox regression with adjustment for total weekly alcohol intake, diabetes, BMI, sex, age, ethnicity, Townsend deprivation index, physical activity, smoke, alcohol type, drinking with/without meals, genotyping array, and first 10 ancestry principal components; p values were calculated using the two-sided Wald test
p=0.653 for multiplicative interaction, assessed through a two-sided likelihood ratio test comparing models with and without interaction terms, between alcohol consumption and polygenic risk group

Supplemental Table 7: Hazard ratios for risk of alcohol-related cirrhosis by diabetes and (a) alcohol consumption and (b) polygenic risk – competing risk regression models (N=312,599)

	ARC (n=734)		
	**HR**	**95% CI**	**p value**
**(a) Alcohol consumption***			
Below daily limit			
Without diabetes	Ref		
With diabetes	2.67	(1.18, 6.01)	0.018
Above daily limit but below binge			
Without diabetes	1.29	(0.90, 1.85)	0.161
With diabetes	3.30	(1.93, 5.64)	<0.001
Binge			
Without diabetes	2.21	(1.55, 3.16)	<0.001
With diabetes	5.43	(3.31, 8.92)	<0.001
Heavy binge			
Without diabetes	3.43	(2.38, 4.95)	<0.001
With diabetes	8.53	(5.43, 13.38)	<0.001
**(b) Genetic risk****			
Low PRS			
Without diabetes	Ref		
With diabetes	2.56	(1.26, 5.17)	0.009
Mid PRS			
Without diabetes	1.61	(1.21, 2.15)	0.001
With diabetes	3.86	(2.59, 5.75)	<0.001
High PRS			
Without diabetes	3.44	(2.57, 4.61)	<0.001
With diabetes	8.96	(5.98, 13.43)	<0.001

HRs were derived from Cox regression, with two-sided p values computed using the Wald test

*Adjusted for sex, age, ethnicity, Townsend deprivation index at recruitment, physical activity, smoking, total weekly alcohol intake, beverage type, drinking with/without meal, BMI, PRS group, genotyping array, and first 10 ancestry principal components

p=0.998 for multiplicative interaction, assessed through a two-sided likelihood ratio test comparing models with and without interaction terms, between diabetes and alcohol consumption

**Adjusted for sex, age, ethnicity, Townsend deprivation index at recruitment, physical activity, smoking, total weekly alcohol intake, beverage type, drinking with/without meal, BMI, alcohol consumption group, genotyping array, and first 10 ancestry principal components

p=0.934 for multiplicative interaction, assessed through a two-sided likelihood ratio test comparing models with and without interaction terms, between diabetes and polygenic risk

Supplemental Table 8: Two-way additive interaction of alcohol consumption, PRS group and diabetes – competing risk regression models (N=312,599)

	ARC (n=734)								
	**RERI**	**95% CI**	**p value**	**AP**	**95% CI**	**p value**	**S**	**95% CI**	**p value**
Above daily limit but below binge # Mid PRS *	-0.34	(-1.72, 1.05)	0.632	-0.39	(-1.71, 0.94)	0.567	-0.58	(-10.08, 8.92)	0.745
Above daily limit but below binge # High PRS *	-1.27	(-4.00, 1.46)	0.363	-0.55	(-1.52, 0.42)	0.271	0.51	(0.05, 0.97)	0.036
Binge # Mid PRS *	-0.01	(-1.16, 1.15)	0.989	-0.01	(-0.99, 0.97)	0.989	0.96	(-4.97, 6.88)	0.989
Binge # High PRS *	-1.65	(-4.95, 1.66)	0.329	-0.58	(-1.58, 0.41)	0.248	0.52	(0.08, 0.97)	0.036
Heavy binge # Mid PRS *	2.19	(-0.14, 4.51)	0.065	***0.32***	***(0.03, 0.61)***	***†0.032***	1.60	(0.66, 2.53)	0.211
Heavy binge # High PRS *	5.81	(-0.45, 12.07)	0.069	***0.47***	***(0.22, 0.71)***	***†<0.001***	2.03	(0.92, 3.14)	0.070
Diabetes # Above daily limit but below binge **	-0.61	(-2.68, 1.46)	0.564	-0.40	(-1.81, 1.01)	0.575	0.45	(-0.52, 1.43)	0.272
Diabetes # Binge **	-0.61	(-3.07, 1.84)	0.624	-0.31	(-1.58, 0.96)	0.634	0.62	(-0.40, 1.63)	0.460
Diabetes # Heavy binge **	***4.23***	***(0.19, 8.27)***	***†0.040***	***0.42***	***(0.13, 0.70)***	***†0.004***	1.86	(0.80, 2.92)	0.113
Diabetes # Mid PRS ***	0.67	(-1.28, 2.62)	0.499	0.18	(-0.32, 0.67)	0.479	1.32	(0.16, 2.48)	0.588
Diabetes # High PRS ***	***4.33***	***(0.65, 8.01)***	***†0.021***	***0.44***	***(0.18, 0.69)***	***†0.001***	1.94	(0.91, 2.97)	0.073

Based on hazard ratio estimates derived from Cox regression, with two-sided p values computed using the Wald test
*Adjusted for total weekly alcohol intake, BMI, diabetes, sex, age, ethnicity, Townsend deprivation index, physical activity, smoke, alcohol type, drinking with/without meals, genotyping array, first 10 ancestry principal components
**Adjusted for total weekly alcohol intake, BMI, sex, age, ethnicity, Townsend deprivation index, physical activity, smoke, alcohol type, drinking with/without meals, PRS group, genotyping array, first 10 ancestry principal components
***Adjusted for total weekly alcohol intake, daily alcohol consumption group, BMI, sex, age, ethnicity, Townsend deprivation index, physical activity, smoke, alcohol type, drinking with/without meals, genotyping array, first 10 ancestry principal components
†RERI>0, AP>0 or S>1 indicates significant additive interaction

Supplemental analyses using imputed data

Supplemental Table 9: Hazard ratios for risk of advanced ARLD associated with alcohol consumption and polygenic risk using imputed data (N=342,541)

	ARC (n=873)			AH (n=163)		
	**HR**	**95% CI**	**p value**	**HR**	**95% CI**	**p value**
Alcohol consumption group *						
Below daily limit	Ref			Ref		
Above daily limit but below binge	1.24	(0.93, 1.66)	0.144	1.91	(0.79, 4.61)	0.153
Binge	2.03	(1.52, 2.72)	<0.001	3.66	(1.53, 8.73)	0.004
Heavy binge	3.30	(2.48, 4.39)	<0.001	6.22	(2.63, 14.70)	<0.001
Polygenic risk score category **						
Low group	Ref			Ref		
Middle group	1.59	(1.25, 2.01)	<0.001	1.25	(0.78, 2.01)	0.360
High group	3.69	(2.89, 4.72)	<0.001	1.92	(1.14, 3.22)	0.014

HRs were derived from Cox regression, with two-sided p values computed using the Wald test
*Adjusted for sex, age, ethnicity, Townsend deprivation index at recruitment, physical activity, smoking, total weekly alcohol intake, beverage type, drinking with/without meal, diabetes, and BMI
**Adjusted for sex, age, ethnicity, Townsend deprivation index at recruitment, physical activity, smoking, total weekly alcohol intake, beverage type, drinking with/without meal, daily alcohol consumption group, diabetes, BMI, first 10 principal genetic components, and genotyping array

Supplemental Table 10: Hazard ratios for risk of alcohol-related cirrhosis by alcohol consumption and polygenic risk group using imputed data (N=342,541)

	ARC (n=873)		
	**HR**	**95% CI**	**p value**
Below daily limit			
Low PRS	Ref		
Mid PRS	1.61	(0.67, 3.87)	0.289
High PRS	4.45	(1.82, 10.86)	0.001
Above daily limit but below binge			
Low PRS	1.30	(0.52, 3.23)	0.579
Mid PRS	1.93	(0.85, 4.37)	0.118
High PRS	5.23	(2.29, 11.97)	<0.001
Binge			
Low PRS	2.11	(0.85, 5.20)	0.106
Mid PRS	3.48	(1.53, 7.93)	0.003
High PRS	6.95	(3.05, 15.84)	<0.001
Heavy binge			
Low PRS	3.19	(1.33, 7.64)	0.009
Mid PRS	5.09	(2.26, 11.49)	<0.001
High PRS	11.67	(5.14, 26.46)	<0.001

HRs were derived from Cox regression with adjustment for total weekly alcohol intake, diabetes, BMI, sex, age, ethnicity, Townsend deprivation index, physical activity, smoke, alcohol type, drinking with/without meals, genotyping array, and first 10 ancestry principal components; p values were calculated using the two-sided Wald test
p=0.873 for multiplicative interaction, assessed through a two-sided likelihood ratio test comparing models with and without interaction terms, between alcohol consumption and polygenic risk group

Supplemental Table 11: Hazard ratios for risk of alcohol-related cirrhosis by diabetes and (a) alcohol consumption and (b) polygenic risk using imputed data (N=342,541)

	ARC (n=873)		
	**HR**	**95% CI**	**p value**
**(a) Alcohol consumption***			
Below daily limit			
Without diabetes	Ref		
With diabetes	2.21	(1.05, 4.66)	0.038
Above daily limit but below binge			
Without diabetes	1.18	(0.86, 1.61)	0.305
With diabetes	2.99	(1.85, 4.82)	<0.001
Binge			
Without diabetes	1.90	(1.39, 2.59)	<0.001
With diabetes	4.19	(2.63, 6.67)	<0.001
Heavy binge			
Without diabetes	2.92	(2.15, 3.98)	<0.001
With diabetes	6.70	(4.49, 9.98)	<0.001
**(b) Genetic risk****			
Low PRS			
Without diabetes	Ref		
With diabetes	2.18	(1.08, 4.37)	0.029
Mid PRS			
Without diabetes	1.59	(1.24, 2.05)	<0.001
With diabetes	3.41	(2.36, 4.92)	<0.001
High PRS			
Without diabetes	3.62	(2.79, 4.69)	<0.001
With diabetes	9.14	(6.37, 13.11)	<0.001

HRs were derived from Cox regression, with two-sided p values computed using the Wald test
*Adjusted for sex, age, ethnicity, Townsend deprivation index at recruitment, physical activity, smoking, total weekly alcohol intake, beverage type, drinking with/without meal, BMI, PRS group, genotyping array, and first 10 ancestry principal components
p=0.969 for multiplicative interaction, assessed through a two-sided likelihood ratio test comparing models with and without interaction terms, between diabetes and alcohol consumption
**Adjusted for sex, age, ethnicity, Townsend deprivation index at recruitment, physical activity, smoking, total weekly alcohol intake, beverage type, drinking with/without meal, BMI, alcohol consumption group, genotyping array, and first 10 ancestry principal components
p=0.725 for multiplicative interaction, assessed through a two-sided likelihood ratio test comparing models with and without interaction terms, between diabetes and polygenic risk

Supplemental Table 12: Two-way additive interaction of alcohol consumption, PRS group and diabetes using imputed data (N=342,541)

	ARC (n=873)								
	**RERI**	**95% CI**	**p value**	**AP**	**95% CI**	**p value**	**S**	**95% CI**	**p value**
Above daily limit but below binge # Mid PRS *	-0.40	(-1.73, 0.93)	0.554	-0.42	(-1.57, 0.73)	0.477	-0.11	(-2.95, 2.74)	0.446
Above daily limit but below binge # High PRS *	-1.08	(-3.51, 1.34)	0.381	-0.42	(-1.23, 0.39)	0.310	0.59	(0.13, 1.06)	0.085
Binge # Mid PRS *	-0.34	(-1.65, 0.97)	0.609	-0.29	(-1.24, 0.66)	0.554	0.35	(-0.62, 1.32)	0.190
Binge # High PRS *	-1.93	(-5.07, 1.21)	0.228	-0.73	(-1.73, 0.26)	0.147	0.46	(0.09, 0.82)	0.004
Heavy binge # Mid PRS *	1.87	(-0.03, 3.77)	0.054	***0.29***	***(0.01, 0.57)***	***†0.039***	1.53	(0.70, 2.36)	0.208
Heavy binge # High PRS *	***6.09***	***(0.74, 11.43)***	***†0.026***	***0.48***	***(0.28, 0.68)***	***†<0.001***	***2.08***	***(1.09, 3.07)***	***†0.032***
Diabetes # Above daily limit but below binge **	-0.33	(-2.01, 1.36)	0.704	-0.22	(-1.38, 0.94)	0.710	0.60	(-0.76, 1.96)	0.560
Diabetes # Binge **	-0.45	(-2.41, 1.50)	0.649	-0.26	(-1.39, 0.88)	0.658	0.63	(-0.46, 1.72)	0.505
Diabetes # Heavy binge **	***3.16***	***(0.23, 6.09)***	***†0.034***	***0.39***	***(0.12, 0.66)***	***†0.005***	1.81	(0.82, 2.80)	0.109
Diabetes # Mid PRS ***	0.61	(-1.07, 2.28)	0.477	0.18	(-0.30, 0.66)	0.457	1.35	(0.12, 2.58)	0.576
Diabetes # High PRS ***	***4.66***	***(1.28, 8.04)***	***†0.007***	***0.46***	***(0.24, 0.69)***	***†<0.001***	***2.05***	***(1.04, 3.07)***	***†0.041***

Based on hazard ratio estimates derived from Cox regression, with two-sided p values computed using the Wald test
*Adjusted for total weekly alcohol intake, BMI, diabetes, sex, age, ethnicity, Townsend deprivation index, physical activity, smoke, alcohol type, drinking with/without meals, genotyping array, first 10 ancestry principal components
**Adjusted for total weekly alcohol intake, BMI, sex, age, ethnicity, Townsend deprivation index, physical activity, smoke, alcohol type, drinking with/without meals, PRS group, genotyping array, first 10 ancestry principal components
***Adjusted for total weekly alcohol intake, daily alcohol consumption group, BMI, sex, age, ethnicity, Townsend deprivation index, physical activity, smoke, alcohol type, drinking with/without meals, genotyping array, first 10 ancestry principal components
†RERI>0, AP>0 or S>1 indicates significant additive interaction

Supplemental Table 13: Three-way additive interaction of alcohol consumption, PRS group and diabetes using imputed data (N=342,541)

	ARC (n=873)		
	**HR**	**95% CI**	**p value**
Below daily limit # Low PRS # No diabetes	Ref		
Heavy binge # Low PRS # No diabetes	2.78	(1.15, 6.74)	0.023
Below daily limit # High PRS # No diabetes	3.71	(1.48, 9.27)	0.005
Below daily limit # Low PRS # Diabetes	1.78	(0.01, 14.87)	0.716
Heavy binge # High PRS # Diabetes	21.62	(8.71, 53.64)	<0.001
	**Total RERI**	**95% CI**	**p value**
Heavy binge # High PRS # Diabetes	***20.37***	***(1.38, 39.36)***	***†0.036***
	**AP**	**95% CI**	**p value**
Heavy binge # High PRS # Diabetes	***0.82***	***(0.69, 0.95)***	***†<0.001***
	**S**	**95% CI**	**p value**
Heavy binge # High PRS # Diabetes	6.97	(0.02, 13.92)	0.092
	**RERI beyond two-way interactions**	**95% CI**	**p value**
Heavy binge # High PRS # Diabetes	-5.57	(-25.27, 14.14)	0.580

HRs were derived from Cox regression with adjustment for total weekly alcohol intake, BMI, sex, age, ethnicity, Townsend deprivation index, physical activity, smoke, alcohol type, drinking with/without meals, genotyping array, first 10 ancestry principal components

RERI, AP, and S were computed based on HRs obtained from Cox regression with aforementioned adjustment

All p values were calculated using the two-sided Wald test

†RERI>0, AP>0 or S>1 indicates significant additive interaction

Replication analyses in the CKB cohort

Supplemental Table 14a: Baseline characteristics of 69,039 male, current weekly drinkers in the China Kadoorie Biobank by disease status

	ARLD-free controls (n=68,807)	ARC (n=191)	AH (n=53)
**Age, years***	51.6 (10.2)	51.8 (9.4)	48.3 (8.7)
**Alcohol intake on a typical day, g per day***	54.2 (36.9)	83.8 (56.7)	80.4 (58.3)
**Weekly alcohol intake, g per week***	284.7 (244.2)	518.5 (376.4)	486.8 (397.8)
**Alcohol consumption group****			
Within daily limit	22,547 (32.8%)	28 (14.7%)	8 (15.1%)
Above daily limit but below binge	29,089 (42.3%)	69 (36.1%)	19 (35.8%)
Binge	8,081 (11.7%)	24 (12.6%)	9 (17.0%)
Heavy Binge	9,090 (13.2%)	70 (36.6%)	17 (32.1%)
**Smoking status**			
Never	5,485 (8.0%)	5 (2.6%)	2 (3.8%)
Occasional or current	54,611 (79.4%)	167 (87.4%)	47 (88.7%)
Ex-regular	8,711 (12.7%)	19 (9.9%)	4 (7.5%)
**Physical activity, MET-h/d*****	22.9 (15.0)	22.8 (14.2)	22.3 (13.7)
**Frequency of drinking**			
1-2 days/week	13,771 (20.0%)	12 (6.3%)	4 (7.5%)
3-5 days/week	12,423 (18.1%)	14 (7.3%)	6 (11.3%)
Daily or almost every day	42,613 (61.9%)	165 (86.4%)	43 (81.1%)
**Alcohol type**			
Beer	12,519 (18.2%)	11 (5.8%)	7 (13.2%)
Rice wine or grape wine	8,363 (12.2%)	17 (8.9%)	3 (5.7%)
Spirits	47,925 (69.7%)	163 (85.3%)	43 (81.1%)
**Drinking with meals**			
Usually drink with the meal	59,138 (85.9%)	140 (73.3%)	45 (84.9%)
Usually drink between or after the meals	5,754 (8.4%)	7 (3.7%)	2 (3.8%)
No regular pattern	3,915 (5.7%)	44 (23.0%)	6 (11.3%)
**BMI, kg/m2**			
<18.5	2,249 (3.3%)	16 (8.4%)	2 (3.8%)
18.5-24.9	43,401 (63.1%)	143 (74.9%)	46 (86.8%)
25-29.9	20,880 (30.3%)	30 (15.7%)	5 (9.4%)
30+	2,277 (3.3%)	2 (1.0%)	0 (0.0%)
**Prevalent diabetes (self-reported or screen-detected)**	3,430 (5.0%)	13 (6.8%)	4 (7.5%)

Values are numbers (percentages) unless otherwise indicated
* Mean (standard deviation)
** Based on average daily alcohol consumption: within daily limit (<32g for men), above daily limit but below binge (32-64g for men), binge (64-96g for men) and heavy binge (>=96g for men)
*** Physical activity calculated as metabolic equivalent task hours per day (MET-h/d)

Supplemental Table 14b: Baseline characteristics of 69,039 male, current weekly drinkers in the China Kadoorie Biobank by alcohol consumption group*

	Total (n=69,039)	Within daily limit (n=22,582)	Above daily limit but below binge (n=29,172)	Binge (n=8,113)	Heavy Binge (n=9,172)	p value ****
**Age, years****	51.6 (10.2)	53.9 (10.9)	50.8 (9.8)	49.9 (9.7)	50.0 (9.5)	<0.001
**Alcohol intake on a typical day, g per day****	54.3 (37.0)	21.2 (6.8)	50.1 (9.7)	80.5 (5.1)	126.0 (37.0)	<0.001
**Weekly alcohol intake, g per week****	285.4 (245.0)	100.8 (59.1)	256.0 (114.5)	434.6 (151.4)	701.6 (319.1)	<0.001
**Smoking status**						
Never	5,492 (8.0%)	2,488 (11.0%)	2,121 (7.3%)	440 (5.4%)	443 (4.8%)	<0.001
Occasional or current	54,813 (79.4%)	16,418 (72.7%)	23,643 (81.0%)	6,820 (84.1%)	7,932 (86.5%)	
Ex-regular	8,734 (12.7%)	3,676 (16.3%)	3,408 (11.7%)	853 (10.5%)	797 (8.7%)	
**Physical activity, MET-h/d**	22.9 (15.0)	21.6 (15.1)	23.2 (14.8)	24.7 (15.2)	23.9 (15.0)	<0.001
**Frequency of drinking**						
1-2 days/week	13,786 (20.0%)	5,873 (26.0%)	5,568 (19.1%)	1,159 (14.3%)	1,186 (12.9%)	<0.001
3-5 days/week	12,443 (18.0%)	4,506 (20.0%)	5,401 (18.5%)	1,295 (16.0%)	1,241 (13.5%)	
Daily or almost every day	42,810 (62.0%)	12,203 (54.0%)	18,203 (62.4%)	5,659 (69.8%)	6,745 (73.5%)	
**Alcohol type**						
Beer	12,536 (18.2%)	6,228 (27.6%)	5,075 (17.4%)	722 (8.9%)	511 (5.6%)	<0.001
Rice wine or grape wine	8,381 (12.1%)	5,487 (24.3%)	2,251 (7.7%)	355 (4.4%)	288 (3.1%)	
Spirits	48,122 (69.7%)	10,867 (48.1%)	21,846 (74.9%)	7,036 (86.7%)	8,373 (91.3%)	
**Drinking with meals**						
Usually drink with the meal	59,314 (85.9%)	19,566 (86.6%)	25,027 (85.8%)	7,227 (89.1%)	7,494 (81.7%)	<0.001
Usually drink between or after the meals	5,763 (8.3%)	1,916 (8.5%)	2,558 (8.8%)	508 (6.3%)	781 (8.5%)	
No regular pattern	3,962 (5.7%)	1,100 (4.9%)	1,587 (5.4%)	378 (4.7%)	897 (9.8%)	
**BMI, kg/m2**						
<18.5	2,267 (3.3%)	951 (4.2%)	872 (3.0%)	187 (2.3%)	257 (2.8%)	<0.001
18.5-24.9	43,579 (63.1%)	14,314 (63.4%)	18,263 (62.6%)	5,145 (63.4%)	5,857 (63.9%)	
25-29.9	20,914 (30.3%)	6,744 (29.9%)	9,095 (31.2%)	2,416 (29.8%)	2,659 (29.0%)	
30+	2,279 (3.3%)	573 (2.5%)	942 (3.2%)	365 (4.5%)	399 (4.4%)	
**Prevalent diabetes (self-reported or screen-detected)**	3,446 (5.0%)	1,194 (5.3%)	1,418 (4.9%)	355 (4.4%)	479 (5.2%)	0.005
**Advanced ARLD**						
ARC or AH	232 (0.3%)	35 (0.2%)	83 (0.3%)	32 (0.4%)	82 (0.9%)	<0.001

ARC	191 (0.3%)	28 (0.1%)	69 (0.2%)	24 (0.3%)	70 (0.8%)	<0.001
AH	53 (0.1%)	8 (0.0%)	19 (0.1%)	9 (0.1%)	17 (0.2%)	<0.001

Values are numbers (percentages) unless otherwise indicated

* Based on average daily alcohol consumption: within daily limit (<32g for men), above daily limit but below binge (32-64g for men), binge (64-96g for men) and heavy binge (>=96g for men)

** Mean (standard deviation)

*** Physical activity calculated as metabolic equivalent task hours per day (MET-h/d)

**** Two-sided p values from one-way ANOVA or chi-square test to compare the differences across binge groups

Supplemental Table 15: Hazard ratios for risk of advanced ARLD associated with alcohol consumption – replication analyses in the CKB (male weekly drinkers, N=69,039)

	AH (n=53)			ARC (n=191)		
	HR	95% CI	p value	HR	95% CI	p value
Alcohol consumption group						
Below daily limit	Ref			Ref		
Above daily limit but below binge	1.41	(0.58, 3.41)	0.443	1.53	(0.96, 2.45)	0.076
Binge	2.39	(0.84, 6.83)	0.103	1.96	(1.08, 3.57)	0.028
Heavy binge	2.67	(0.92, 7.69)	0.069	2.91	(1.67, 5.10)	<0.001

HRs were derived from Cox regression with adjustment for age, study areas, education, physical activity, smoking, total weekly alcohol intake, beverage type, drinking with/without meal, diabetes, and BMI; p values were calculated using the two-sided Wald test

Supplemental Table 16: Hazard ratios for risk ARC by diabetes and alcohol consumption – replication analyses in the CKB (male weekly drinkers, N=69,039)

	HR	95% CI	p value
**ARC (n=191)**			
Below binge*			
Without diabetes	Ref		
With diabetes	1.56	(0.62, 3.87)	0.343
Binge			
Without diabetes	1.42	(0.88, 2.30)	0.153
With diabetes	2.16	(0.30, 15.63)	0.446
Heavy binge			
Without diabetes	1.95	(1.29, 2.95)	0.001
With diabetes	6.69	(2.98, 15.02)	<0.001

HRs were derived from Cox regression with adjustment for age, study areas, education, physical activity, smoking, total weekly alcohol intake, beverage type, drinking with/without meal, diabetes, and BMI; p values were calculated using the two-sided Wald test
p=0.828 for multiplicative interaction, assessed through a two-sided likelihood ratio test comparing models with and without interaction terms, between diabetes and alcohol consumption
*The two groups (below daily limit, above daily limit but below binge) were combined due to zero cases of ARC in participants who reported drinking below daily limit and had diabetes

Supplemental Table 17: Two-way additive interaction of alcohol consumption and diabetes – replication analyses in the CKB (male weekly drinkers, N=69,039)

	RERI	95% CI	p value	AP	95% CI	p value	S	95% CI	p value
**ARC (n=191)**									
Diabetes # Binge*	-0.002	(-2.918, 2.914)	0.999	-0.002	(-2.232, 2.229)	0.999	0.99	(-8.37, 10.36)	0.999
Diabetes # Heavy binge*	4.37	(-1.14, 9.88)	0.120	***0.63***	***(0.29, 0.98)***	***†<0.001***	3.87	(-1.17, 8.90)	0.264

Based on hazard ratio estimates derived from Cox regression with adjustment for age, study areas, education, physical activity, smoking, total weekly alcohol intake, beverage type, drinking with/without meal, diabetes, and BMI; p values were calculated using the two-sided Wald test
*Compared to participants who reported drinking below binge and were free of diabetes
†RERI>0, AP>0 or S>1 indicates significant additive interaction

Supplemental Table 18: Hazard ratios for risk of advanced ARLD associated with per 1 SD increase in PRS in the CKB

	Cases (n)	HR	95% CI	p value
Genotyped males*				
AH (n=30,760)	13	1.13	(0.65, 1.97)	0.656
ARC (n=30,771)	52	1.13	(0.86, 1.48)	0.386
Genotyped male weekly drinkers**				
AH (n=10,737)	10	0.80	(0.42, 1.52)	0.489
ARC (n=10,745)	41	1.08	(0.79, 1.48)	0.626

Analyses were based on randomly-selected genotyped men (n=30,756 men without prior cirrhosis/hepatitis) and, to increase study power, additional recorded liver disease cases selected from a subset enriched by CVD/COPD cases (4 additional AH cases, 15 additional ARC cases); HRs were derived from Cox regression, with two-sided p values computed using the Wald test

*Adjusted for age, study areas, education, smoking, BMI, physical activity, diabetes, alcohol consumption group (never, former, occasional, monthly, reduced intake, and weekly drinkers), 11 genomic PCs

**Adjusted for age, study areas, education, smoking, BMI, physical activity, diabetes, total weekly alcohol intake, beverage type, drinking with/without meal, daily alcohol consumption group, 11 genomic PCs

Cross-validation analyses using UKB sub-samples

Supplemental Table 19: Hazard ratios for risk of advanced ARLD associated with alcohol consumption and polygenic risk – cross-validation using sub-samples

	ARC			AH		
	**HR**	**95% CI**	**p value**	**HR**	**95% CI**	**p value**
**Sub-sample #1 (N=156303, ARC=362, AH=71)**						
Alcohol consumption group *						
Below daily limit	Ref			Ref		
Above daily limit but below binge	1.31	(0.81, 2.12)	0.271	1.89	(0.41, 8.80)	0.416
Binge	2.40	(1.50, 3.86)	<0.001	5.76	(1.32, 25.03)	0.020
Heavy binge	3.83	(2.39, 6.12)	<0.001	9.47	(2.19, 41.00)	0.003
Polygenic risk score category **						
Low group	Ref			Ref		
Middle group	1.84	(1.26, 2.68)	0.002	1.32	(0.65, 2.69)	0.450
High group	3.85	(2.62, 5.67)	<0.001	1.42	(0.64, 3.14)	0.393
**Sub-sample #2 (N=156296, ARC=372, AH=67)**						
Alcohol consumption group *						
Below daily limit	Ref			Ref		
Above daily limit but below binge	1.34	(0.86, 2.11)	0.199	2.84	(0.64, 12.54)	0.169
Binge	2.28	(1.46, 3.57)	<0.001	4.47	(1.01, 19.73)	0.048
Heavy binge	3.67	(2.35, 5.71)	<0.001	8.91	(2.07, 38.43)	0.003
Polygenic risk score category **						
Low group	Ref			Ref		
Middle group	1.59	(1.11, 2.29)	0.012	1.14	(0.54, 2.37)	0.732
High group	3.52	(2.43, 5.09)	<0.001	1.97	(0.90, 4.31)	0.090

HRs were derived from Cox regression, with two-sided p values computed using the Wald test
*Adjusted for sex, age, ethnicity, Townsend deprivation index at recruitment, physical activity, smoking, total weekly alcohol intake, beverage type, drinking with/without meal, diabetes, and BMI
**Adjusted for sex, age, ethnicity, Townsend deprivation index at recruitment, physical activity, smoking, total weekly alcohol intake, beverage type, drinking with/without meal, daily alcohol consumption group, diabetes, BMI, first 10 principal genetic components, and genotyping array

Supplemental Table 20: Hazard ratios for risk of alcohol-related cirrhosis by alcohol consumption and polygenic risk group – cross-validation using sub-samples

	Sub-sample #1 (N=156303, ARC=362)			Sub-sample #2 (N=156296, ARC=372)		
	**HR**	**95% CI**	**p value**	**HR**	**95% CI**	**p value**
Below daily limit						
Low PRS	Ref			Ref		
Mid PRS	4.04	(0.52, 31.30)	0.182	0.91	(0.29, 2.86)	0.870
High PRS	11.55	(1.48, 90.33)	0.020	2.56	(0.80, 8.18)	0.112
Above daily limit but below binge						
Low PRS	3.04	(0.37, 24.76)	0.298	1.20	(0.39, 3.74)	0.748
Mid PRS	4.79	(0.65, 35.11)	0.123	1.25	(0.45, 3.52)	0.669
High PRS	13.55	(1.85, 99.47)	0.010	3.10	(1.09, 8.82)	0.033
Binge						
Low PRS	6.98	(0.90, 54.19)	0.063	1.00	(0.29, 3.43)	0.999
Mid PRS	9.95	(1.37, 72.27)	0.023	2.51	(0.91, 6.97)	0.077
High PRS	18.46	(2.52, 135.38)	0.004	4.55	(1.61, 12.85)	0.004
Heavy binge						
Low PRS	7.20	(0.93, 56.08)	0.059	1.85	(0.59, 5.80)	0.289
Mid PRS	15.57	(2.15, 112.54)	0.007	3.40	(1.23, 9.40)	0.019
High PRS	29.55	(4.07, 214.69)	0.001	7.71	(2.78, 21.37)	<0.001

HRs were derived from Cox regression with adjustment for total weekly alcohol intake, diabetes, BMI, sex, age, ethnicity, Townsend deprivation index, physical activity, smoke, alcohol type, drinking with/without meals, genotyping array, and first 10 ancestry principal components; p values were calculated using the two-sided Wald test

Supplemental Table 21: Hazard ratios for risk of alcohol-related cirrhosis by diabetes and (a) alcohol consumption and (b) polygenic risk – cross-validation using sub-samples

	Sub-sample #1 (N=156303, ARC=362)			Sub-sample #2 (N=156296, ARC=372)		
	**HR**	**95% CI**	**p value**	**HR**	**95% CI**	**p value**
**(a) Alcohol consumption***						
Below daily limit						
Without diabetes	Ref					
With diabetes	3.65	(1.23, 10.86)	0.020	2.16	(0.64, 7.27)	0.212
Above daily limit but below binge						
Without diabetes	1.36	(0.80, 2.30)	0.254	1.22	(0.76, 1.99)	0.411
With diabetes	3.19	(1.42, 7.17)	0.005	3.75	(1.84, 7.65)	<0.001
Binge						
Without diabetes	2.42	(1.44, 4.06)	0.001	2.01	(1.24, 3.24)	0.004
With diabetes	5.75	(2.76, 11.96)	<0.001	5.61	(2.83, 11.13)	<0.001
Heavy binge						
Without diabetes	3.67	(2.19, 6.17)	<0.001	3.03	(1.88, 4.88)	<0.001
With diabetes	8.81	(4.63, 16.75)	<0.001	8.83	(4.85, 16.09)	<0.001
**(b) Genetic risk****						
Low PRS						
Without diabetes	Ref					
With diabetes	6.02	(2.68, 13.55)	<0.001	0.55	(0.08, 4.06)	0.562
Mid PRS						
Without diabetes	2.25	(1.46, 3.46)	<0.001	1.38	(0.95, 2.00)	0.089
With diabetes	3.85	(2.07, 7.17)	<0.001	4.82	(2.92, 7.96)	<0.001
High PRS						
Without diabetes	4.42	(2.85, 6.87)	<0.001	3.17	(2.17, 4.64)	<0.001
With diabetes	12.47	(6.98, 22.29)	<0.001	8.57	(4.99, 14.73)	<0.001

HRs were derived from Cox regression, with two-sided p values computed using the Wald test
*Adjusted for sex, age, ethnicity, Townsend deprivation index at recruitment, physical activity, smoking, total weekly alcohol intake, beverage type, drinking with/without meal, BMI, PRS group, genotyping array, and first 10 ancestry principal components
**Adjusted for sex, age, ethnicity, Townsend deprivation index at recruitment, physical activity, smoking, total weekly alcohol intake, beverage type, drinking with/without meal, BMI, alcohol consumption group, genotyping array, and first 10 ancestry principal components

Supplemental Table 22: Two-way additive interaction of alcohol consumption, PRS group and diabetes – cross-validation using sub-samples

	RERI	95% CI	p value	AP	95% CI	p value	S	95% CI	p value
**Sub-sample #1 (N=156303, ARC=362)**									
Heavy binge # High PRS *	15.53	(-17.20, 48.25)	0.352	***0.40***	***(0.10, 0.70)***	***†0.010***	1.70	(0.79, 2.60)	0.131
Diabetes # Heavy binge **	3.00	(-2.72, 8.72)	0.304	0.30	(-0.17, 0.78)	0.212	1.51	(0.33, 2.68)	0.400
Diabetes # High PRS ***	2.59	(-3.52, 8.71)	0.405	0.24	(-0.24, 0.72)	0.337	1.35	(0.40, 2.30)	0.471
**Sub-sample #2 (N=156296, ARC=372)**									
Heavy binge # High PRS *	0.51	(-1.74, 2.77)	0.656	0.16	(-0.57, 0.89)	0.660	1.31	(-0.58, 3.21)	0.745
Diabetes # Heavy binge **	***6.22***	***(0.08, 12.36)***	***†0.047***	***0.55***	***(0.23, 0.87)***	***†0.001***	2.52	(0.34, 4.69)	0.171
Diabetes # High PRS ***	***5.92***	***(1.61, 10.23)***	***†0.007***	***0.69***	***(0.49, 0.89)***	***†<0.001***	4.54	(0.15, 8.92)	0.114

Based on hazard ratio estimates derived from Cox regression, with two-sided p values computed using the Wald test
*Adjusted for total weekly alcohol intake, BMI, diabetes, sex, age, ethnicity, Townsend deprivation index, physical activity, smoke, alcohol type, drinking with/without meals, genotyping array, first 10 ancestry principal components
**Adjusted for total weekly alcohol intake, BMI, sex, age, ethnicity, Townsend deprivation index, physical activity, smoke, alcohol type, drinking with/without meals, PRS group, genotyping array, first 10 ancestry principal components
***Adjusted for total weekly alcohol intake, daily alcohol consumption group, BMI, sex, age, ethnicity, Townsend deprivation index, physical activity, smoke, alcohol type, drinking with/without meals, genotyping array, first 10 ancestry principal components
†RERI>0, AP>0 or S>1 indicates significant additive interaction

Supplemental Table 23: Three-way additive interaction of alcohol consumption, PRS group and diabetes – cross-validation using sub-samples

	Sub-sample #1 (N=156303, ARC=362)			Sub-sample #2 (N=156296, ARC=372)		
	**HR**	**95% CI**	**p value**	**HR**	**95% CI**	**p value**
Below daily limit # Low PRS # No diabetes	Ref			Ref		
Heavy binge # Low PRS # No diabetes	4.87	(0.61, 39.23)	0.137	1.85	(0.59, 5.81)	0.289
Below daily limit # High PRS # No diabetes	8.25	(1.01, 67.08)	0.049	2.45	(0.75, 7.97)	0.136
Below daily limit # Low PRS # Diabetes	1.18	(0.13, 10.54)	0.880	2.19	(0.59, 8.15)	0.243
Heavy binge # High PRS # Diabetes	65.47	(8.29, 517.10)	<0.001	15.98	(4.93, 51.78)	<0.001
	**Total RERI**	**95% CI**	**p value**	**Total RERI**	**95% CI**	**p value**
Heavy binge # High PRS # Diabetes	69.63	(-76.80, 216.06)	0.351	5.69	(-1.29, 12.67)	0.110
	**AP**	**95% CI**	**p value**	**AP**	**95% CI**	**p value**
Heavy binge # High PRS # Diabetes	***0.82***	***(0.66, 0.98)***	***†<0.001***	***0.80***	***(0.41, 1.20)***	***†<0.001***
	**S**	**95% CI**	**p value**	**S**	**95% CI**	**p value**
Heavy binge # High PRS # Diabetes	5.82	(0.26, 11.39)	0.090	15.27	(-114.68, 145.23)	0.830
	**RERI beyond two-way interactions**	**95% CI**	**p value**	**RERI beyond two-way interactions**	**95% CI**	**p value**
Heavy binge # High PRS # Diabetes	-40.53	(-167.10, 86.05)	0.530	1.28	(-10.72, 13.28)	0.834

HRs were derived from Cox regression with adjustment for total weekly alcohol intake, BMI, sex, age, ethnicity, Townsend deprivation index, physical activity, smoke, alcohol type, drinking with/without meals, genotyping array, first 10 ancestry principal components
RERI, AP, and S were computed based on HRs obtained from Cox regression with aforementioned adjustment
All p values were calculated using the two-sided Wald test
†RERI>0, AP>0 or S>1 indicates significant additive interaction
