# Supplementary material for: Binge-pattern alcohol consumption and genetic risk as determinants of alcohol-related liver disease
Source: Nat Commun. 2023 Dec 14;14:8041. doi: 10.1038/s41467-023-43064-x (PMC10721893; doi:10.1038/s41467-023-43064-x)
Supplement: Supplementary file 3 — Reporting Summary [file 41467_2023_43064_MOESM3_ESM.pdf]

nature portfolio

Corresponding author(s): Bell, Mehta

Last updated by author(s): Oct 17, 2023

Reporting Summary

Nature Portfolio wishes to improve the reproducibility of the work that we publish. This form provides structure for consistency and transparency in reporting. For further information on Nature Portfolio policies, see our Editorial Policies and the Editorial Policy Checklist.

Statistics

For all statistical analyses, confirm that the following items are present in the figure legend, table legend, main text, or Methods section.

n/a	Confirmed	
☐	☒	The exact sample size (n) for each experimental group/condition, given as a discrete number and unit of measurement
☐	☒	A statement on whether measurements were taken from distinct samples or whether the same sample was measured repeatedly
☐	☒	The statistical test(s) used AND whether they are one- or two-sided<br>*Only common tests should be described solely by name; describe more complex techniques in the Methods section.*
☐	☒	A description of all covariates tested
☐	☒	A description of any assumptions or corrections, such as tests of normality and adjustment for multiple comparisons
☐	☒	A full description of the statistical parameters including central tendency (e.g. means) or other basic estimates (e.g. regression coefficient) AND variation (e.g. standard deviation) or associated estimates of uncertainty (e.g. confidence intervals)
☐	☒	For null hypothesis testing, the test statistic (e.g. F, t, r) with confidence intervals, effect sizes, degrees of freedom and P value noted<br>*Give P values as exact values whenever suitable.*
☒	☐	For Bayesian analysis, information on the choice of priors and Markov chain Monte Carlo settings
☒	☐	For hierarchical and complex designs, identification of the appropriate level for tests and full reporting of outcomes
☒	☐	Estimates of effect sizes (e.g. Cohen's d, Pearson's r), indicating how they were calculated

Our web collection on statistics for biologists contains articles on many of the points above.

Software and code

Policy information about availability of computer code

Data collection	Data were retrieved using Application Program Interfaces provided by UKB and CKB.
Data analysis	All statistical analyses were performed in R, version 4.0.5.

For manuscripts utilizing custom algorithms or software that are central to the research but not yet described in published literature, software must be made available to editors and reviewers. We strongly encourage code deposition in a community repository (e.g. GitHub). See the Nature Portfolio guidelines for submitting code & software for further information.

Data

Policy information about availability of data

All manuscripts must include a data availability statement. This statement should provide the following information, where applicable:

- Accession codes, unique identifiers, or web links for publicly available datasets
- A description of any restrictions on data availability
- For clinical datasets or third party data, please ensure that the statement adheres to our policy

All data provided from the UKB are available to other investigators online upon permission granted by www.ukbiobank.ac.uk. Restrictions apply to the availability of these data, which were used under license for the current study (application ID 42520). The individual-level data on PRS and ARLD-related hospitalization generated in this study have been deposited in the UKB repository under upload ID 4475 and can be accessed with permission from the UKB. Similarly, data from CKB are available to open access users upon permission granted from www.ckbiobank.org. A research proposal will be requested to ensure that any analysis is performed by bona fide researchers and, where data are not currently available to open access researchers, is restricted to the topic covered in this paper. Source data are

provided with this paper.

Research involving human participants, their data, or biological material

Policy information about studies with human participants or human data. See also policy information about sex, gender (identity/presentation), and sexual orientation and race, ethnicity and racism.

Reporting on sex and gender	The UKB collects information on biological sex of all participants, which is self-reported. For the primary analyses in UKB, sex was included as a covariate in all analyses. The validation cohort was derived from the CKB, where information is biological sex is also collected, again by self-report. Analyses in this validation cohort were limited to biological males, due to the very small number of females that regularly drank alcohol.
Reporting on race, ethnicity, or other socially relevant groupings	Ethnicity data was collected in UKB by self-report; ethnicity was categorized into white British, white Irish, other white background, south Asian, black (Caribbean or African), Chinese, mixed or others. Baseline characteristics for our cohort are listed in Table 1 and Supplemental Table 1. Ethnicity was included as a covariate for all analyses in UKB.<br>The validation cohort was drawn from CKB, which is Han Chinese (~97%) by self-report.
Population characteristics	The primary analyses were conducted in 312,599 participants from UKB, who were regular (weekly) alcohol drinkers and complete data sets. Participant characteristics, including relevant covariates, are listed in Table 1 and Supplemental Table 2. Analyses in the validation cohort were conducted in 69,039 male weekly drinkers from CKB. Participant characteristics for this group are listed in Supplemental Tables 14a and 14b.
Recruitment	Recruitment for UKB was through invitation; this took a stratified approach, with over-sampling of some age, sex and deprivation sub-groups. Participation was voluntary and the response rate was 5.5%. Further details are provided in Sudlow et al.; PMID: 25826379. Recruitment for CKB was also through invitation, in 10 geographically defined regions (5 urban and 5 rural) of China, chosen according to local disease patterns, exposure to certain risk factors, population stability, quality of death and disease registries, local commitment and capacity. Further details are provided in Chen et al.; PMID: 22158673.
Ethics oversight	Ethical approval for UKB was covered under NHS National Research Ethics Service (Ref 11/NW/0382). Ethical approval for CKB was from the Ethical Review Committee of the Chinese Centre for Disease Control and Prevention (Beijing, China, 005/2004) ad the Oxford Tropical Research Ethics Committee, University of Oxford (UK, 025-04).

Note that full information on the approval of the study protocol must also be provided in the manuscript.

Field-specific reporting

Please select the one below that is the best fit for your research. If you are not sure, read the appropriate sections before making your selection.

☒ Life sciences ☐ Behavioural & social sciences ☐ Ecological, evolutionary & environmental sciences

For a reference copy of the document with all sections, see nature.com/documents/nr-reporting-summary-flat.pdf

Life sciences study design

All studies must disclose on these points even when the disclosure is negative.

Sample size	The maximum number of participants available for analysis, within our inclusion criteria, were selected from UKB and CKB for analysis.
Data exclusions	As stated in the methods section of the manuscript, only weekly alcohol drinkers were included in the study sample; exclusions were pre-existing liver disease or incomplete data sets. The only further data sets were female drinkers in the CKB validation cohort, since only 2% of female participants reported regular alcohol use.
Replication	The data from CKB represent a validation cohort, to replicate the interactions between binge-pattern drinking, diabetes mellitus and alcohol-related liver disease found in our primary analyses. In this cohort, the following findings were replicated: binge-pattern drinking was independently associated with higher risk of alcohol-related liver disease, and diabetes mellitus had an additive interaction with heavy binge drinking. The findings of an interaction between polygenic risk score and binge-pattern drinking for risk of alcohol-related liver disease were not replicated in CKB. The association of polygenic risk score and alcohol-related liver disease was directionally concordant, but possibly underpowered to completely replicate the primary analyses in UKB.
Randomization	This was a prospectively recruited observational cohort, and thus randomization was not directly relevant to the study design.
Blinding	Disease and hospitalization events were coded by hospital staff blinded to baseline study information. The data provided to researchers did not contain and personally identifiable variables.

Reporting for specific materials, systems and methods

We require information from authors about some types of materials, experimental systems and methods used in many studies. Here, indicate whether each material, system or method listed is relevant to your study. If you are not sure if a list item applies to your research, read the appropriate section before selecting a response.

Materials & experimental systems

n/a	Involved in the study
☒	☐ Antibodies
☒	☐ Eukaryotic cell lines
☒	☐ Palaeontology and archaeology
☒	☐ Animals and other organisms
☒	☐ Clinical data
☒	☐ Dual use research of concern
☒	☐ Plants

Methods

n/a	Involved in the study
☒	☐ ChIP-seq
☒	☐ Flow cytometry
☒	☐ MRI-based neuroimaging
